# Genes, pathways and networks responding to drought stress in oil palm roots

**DOI:** 10.1038/s41598-020-78297-z

**Published:** 2020-12-04

**Authors:** Le Wang, May Lee, Baoqing Ye, Gen Hua Yue

**Affiliations:** 1 Molecular Population Genetics and Breeding Group, Temasek Life Sciences Laboratory, National University of Singapore, 1 Research Link, Singapore, 117604 Singapore; 2 Department of Biological Sciences, National University of Singapore, 14 Science Drive 4, Singapore, 117543 Singapore

**Keywords:** Genetics, Plant sciences

## Abstract

Oil palm is the most productive oilseed crop and its oil yield is seriously affected by frequent drought stress. However, little is known about the molecular responses of oil palm to drought stress. We studied the root transcriptomic responses of oil palm seedlings under 14-day drought stress. We identified 1293 differentially expressed genes (DEGs), involved in several molecular processes, including cell wall biogenesis and functions, phenylpropanoid biosynthesis and metabolisms, ion transport and homeostasis and cellular ketone metabolic process, as well as small molecule biosynthetic process. DEGs were significantly enriched into two categories: hormone regulation and metabolism, as well as ABC transporters. In addition, three protein–protein interaction networks: ion transport, reactive nitrogen species metabolic process and nitrate assimilation, were identified to be involved in drought stress responses. Finally, 96 differentially expressed transcription factors were detected to be associated with drought stress responses, which were classified into 28 families. These results provide not only novel insights into drought stress responses, but also valuable genomic resources to improve drought tolerance of oil palm by both genetic modification and selective breeding.

## Introduction

Oil palm (*Elaeis guineensis*, Jacq.) is the most productive oilseed crop^1^. Palm oil extracted from palm fruits has been used as cooking oil and in making cosmetics, candles, soaps, biofuels, and lubricating greases, as well in processing tinplate and coating iron plates, and the demand for palm oil is increasing^1^. Oil palm is mainly cultivated in the tropical regions of Southeast Asia, Africa and South America^2,3^, where water is particularly important to the productivity of this crop. Oil palm needs ~ 2000 mm/year rain water to have a normal production and does not tolerate to drought for more than 90 days^3^. However, with unpredictable global climate changes, tropical regions are accidentally in danger of lacking of rainfall of up to months^4^. Continuous drought condition affects oil palm production frequently^5^. A previous study showed that deficit of 100 mm water reduced the yield by 20%^6^. Recent studies demonstrated that drought stress for 7–21 days induced physiological and growth changes in oil palm seedlings^7–9^. However, not much is known about transcriptomic response to drought stress. Therefore, it is essential to increase drought tolerance in oil palm to ensure sustainable development of palm oil industry. Understanding of more about the response of oil palm to drought stress will facilitate the breeding of drought tolerant oil palm varieties for sustainable oil production^5,10,11^.

An increasing number of studies have focused on understanding the molecular mechanisms of drought tolerance in agronomic plant species or model plant species (e.g. *Arabidopsis*) and have identified important genes and/or evolutionary conserved genetic elements that act to regulate drought tolerance^12–14^. Drought resistance is complex and determined by genetic and environmental factors, and their interactions. It takes very long time to increase drought tolerance by conventional breeding^15^. The mechanisms underlying drought tolerance are variable among different species^16–18^ and are still poorly understood for most non-model species^19^. Thus, identifying the genetic factors and understanding the underlying mechanisms are essential to improve drought tolerance by gene modifications^20^ and breeding^5^ in different species.

Plants may use diverse mechanisms to maintain water and ion homeostasis^19^. At the molecular levels, many drought-responsive genes and transcription factors have been identified^21^. Some gene classes playing crucial roles in defending against drought stress have been reported^22^. For example, some hormone-related genes can initiate drought resistance signalling^23^. Transporters, like ABC transport system, are essential for osmotic signal and osmosensing mechanism^24^. Endogenous abscisic acid (ABA), which works as an important phytohormone, has well-known functions in drought tolerance in plants, by inducing the expression of stress-related genes^25^. Another study also indicated that jasmonic acid (JA) was associated with stomatal closure to increase drought tolerance^26^. In addition, it was reported that transcription factors (TFs), including NAC, GmNAC, HD-STARTtype and NF-YB family members, played important roles in drought tolerance by regulation of hormone metabolisms^27^. Beyond individual genes, cascaded signalling pathways and regulatory networks were suggested to have played indispensable roles in drought tolerance^28^. ABA coupled with corresponding TFs and ABA responsive elements, could regulate the expressions of a wide range of genes under osmotic stress, via cis regulations^28^. A number of protein families in the calcium signalling pathways, mitogen-activated protein kinases (MAPKs) signalling pathways and phosphorylation cascades were also involved in drought stress responses^29,30^. Previous studies on the responses of plants to drought stress have shed new insights in understanding of the mechanisms of drought tolerance in different species. However, in the oil palm, transcriptomic responses to drought stress are still poorly understood.

The purpose of the current study was to identify genes, pathways, networks and transcription factors involved in drought response using RNA-seq and bioinformatics analysis to understand more about molecular responses of oil palm roots under drought stress. RNA-seq is a next-generation sequencing technology used to analyse the presence and quantity of RNA molecules in biological samples^31^. Herein, oil palm seedlings were firstly treated with drought stress. We studied the transcriptome responses of roots to drought in the primary tissue for stress signal perception and initiating of cascade gene regulation pathways in response to drought. A total of 1293 DEGs, including 96 transcription factors, were identified in drought stress responses. Besides individual genes, signalling pathways and protein–protein interaction networks, involving transcription factors, also likely have played crucial roles in drought tolerance. This study provides both novel insights of molecular response of oil palm to drought stress and genomic resources to improve and develop drought-tolerant oil palms for sustainable oil production.

## Results and discussion

### Morphological and physiological responses to drought

**Figure 1:**
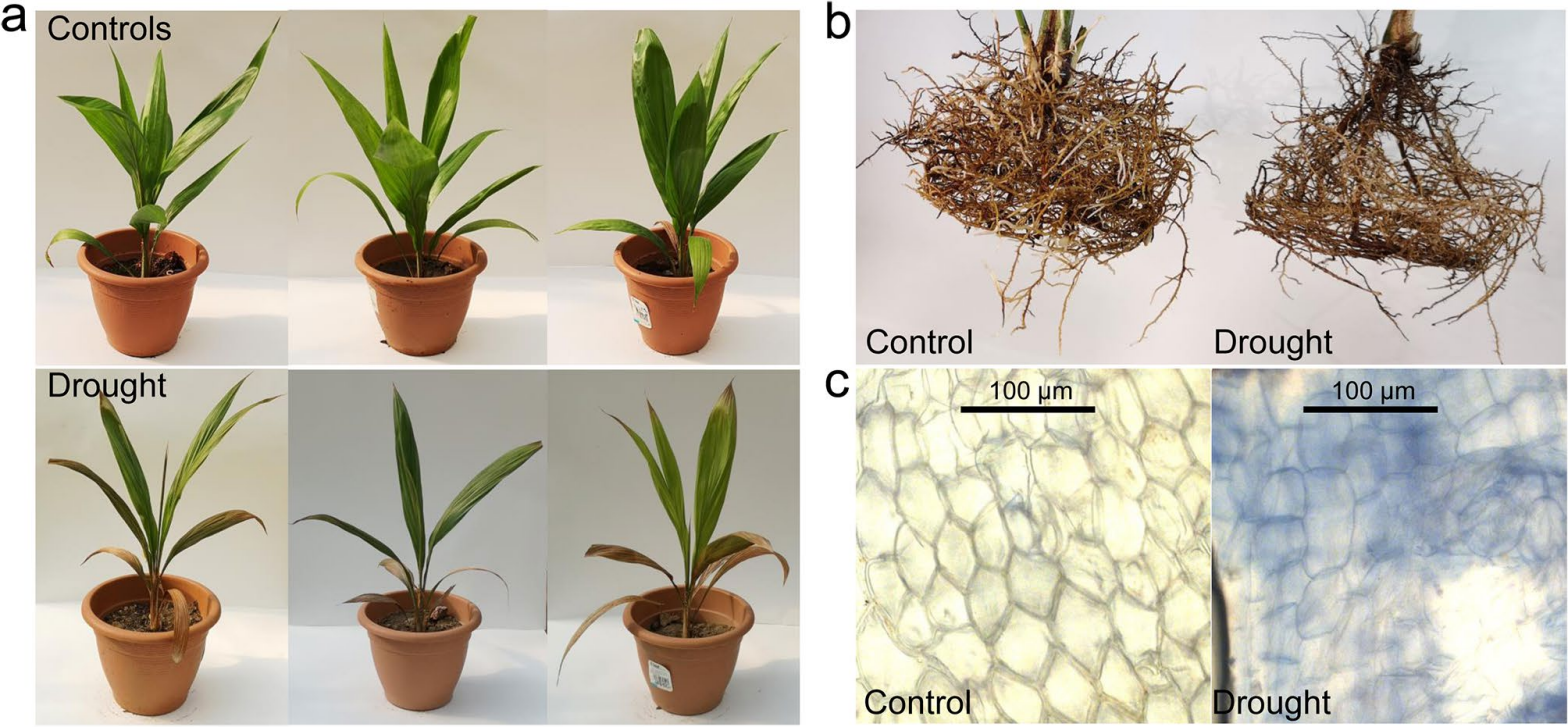
Phenotypic changes of oil palm seedlings to drought stress. The leaves (**a**) and roots (**b**) responses to severe drought stress in comparison to controls at 10 days post challenge, where both leaves and roots were significantly affected by drought, and (**c**) trypan blue staining of roots between control and drought stress groups, where damaged cells were stained.

Obvious morphological changes in leaves and roots of oil palm seedlings under drought stress with a period of 14 days were observed. The effects of drought stress were first observed in leaf morphology, showing initial edge and tip necrosis and then wilting and yellowing for the drought treated samples (Fig. 1a). In comparison to the control, the drought-stressed palms showed significant decrease in the number of roots, root volume and overall biomass (Fig. 1b). Trypan blue staining showed that the drought treatment roots experienced not only obvious cell deformation but also more cell membrane injury than that of the well-watered controls (Fig. 1c). These observations are consistent with those of previous studies in oil palm^7–9^ and other plant species^32–34^ under drought stresses and undergoing water deprivation. These results indicate substantial physiological responses of the oil palm seedlings under drought stress^35^, and provided useful starting materials to study the genes, pathways and networks involved in drought responses using RNA-seq^36^ and bioinformatics analysis.

### Differentially expressed gene (DEGs) in roots of oil palm seedling under drought stress

An average of 70.2 million (M) cleaned reads out of 71.3 M raw reads, were obtained across all six samples. The controls had higher reads coverage than the drought-stressed palms (84.8 M vs 55.7 M) (Supplementary Table S1). Nevertheless, the sequence coverage of the drought-stressed palms (> 200 × of transcriptomes) was sufficient to construct transcripts and identify DEGs. Approximately 70% of cleaned reads were uniquely mapped to the reference genome of 31,640 annotated protein coding genes^37^. The drought stressed seedlings showed slightly higher uniquely mapping rates than the controls (72.1% vs 66.3%), indicating the duplicated genes also play important roles in response to drought stress^38^ in oil palm, a species of palaeotetraploid origin^37^ and future studies should also focus on paralogous genes and their potential functions in stress responses^39^.

**Figure 2:**
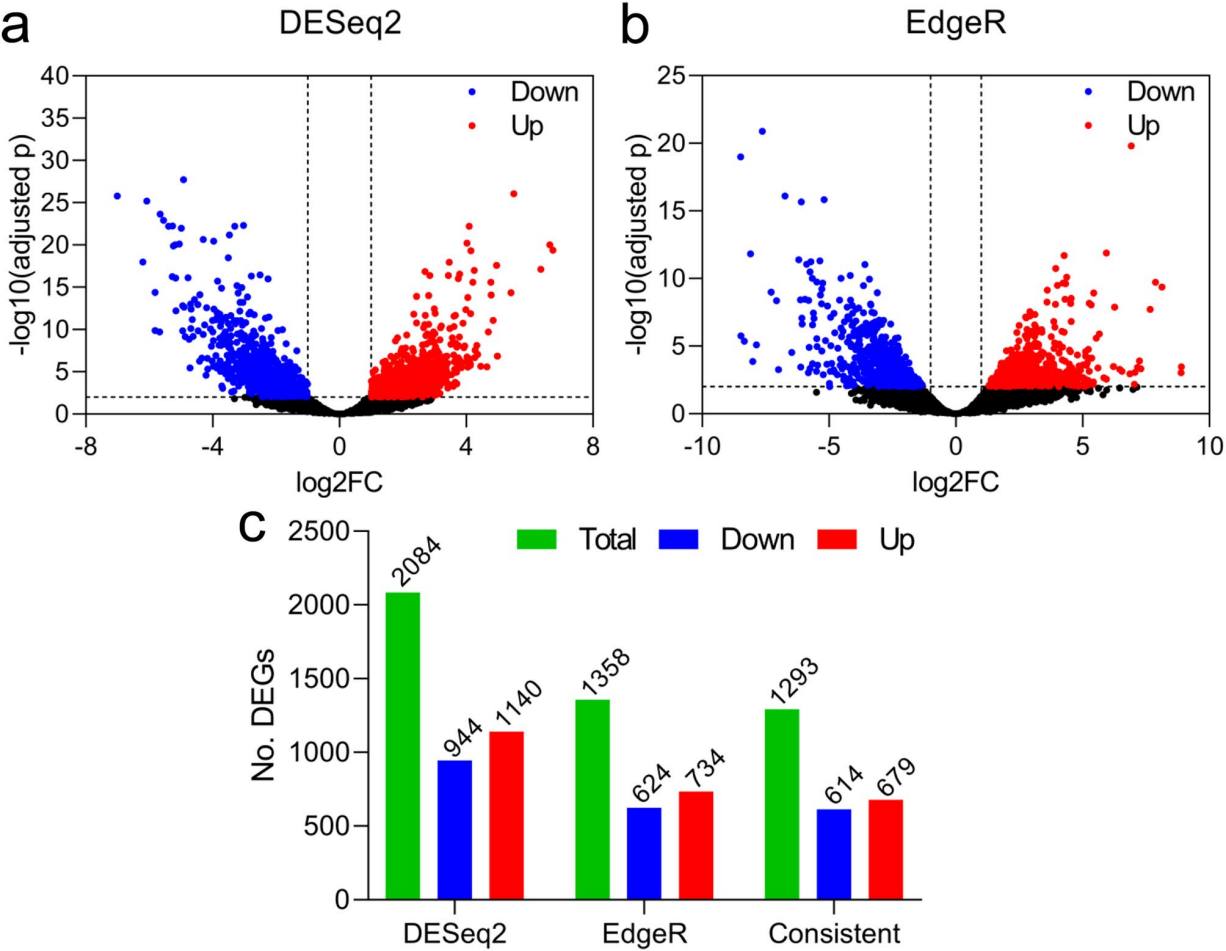
Comparison of differentially expressed genes (DEGs) in the roots of oil palm seedlings under drought stress, as shown by Volcano plot, identified by DESeq2 (**a**) and EdgeR (**b**). The numbers of DEGs that are down-regulated and up-regulated revealed by DESeq2 and EdgeR and the consistent DEGs between the two methods are shown in (**c**).

**Figure 3:**
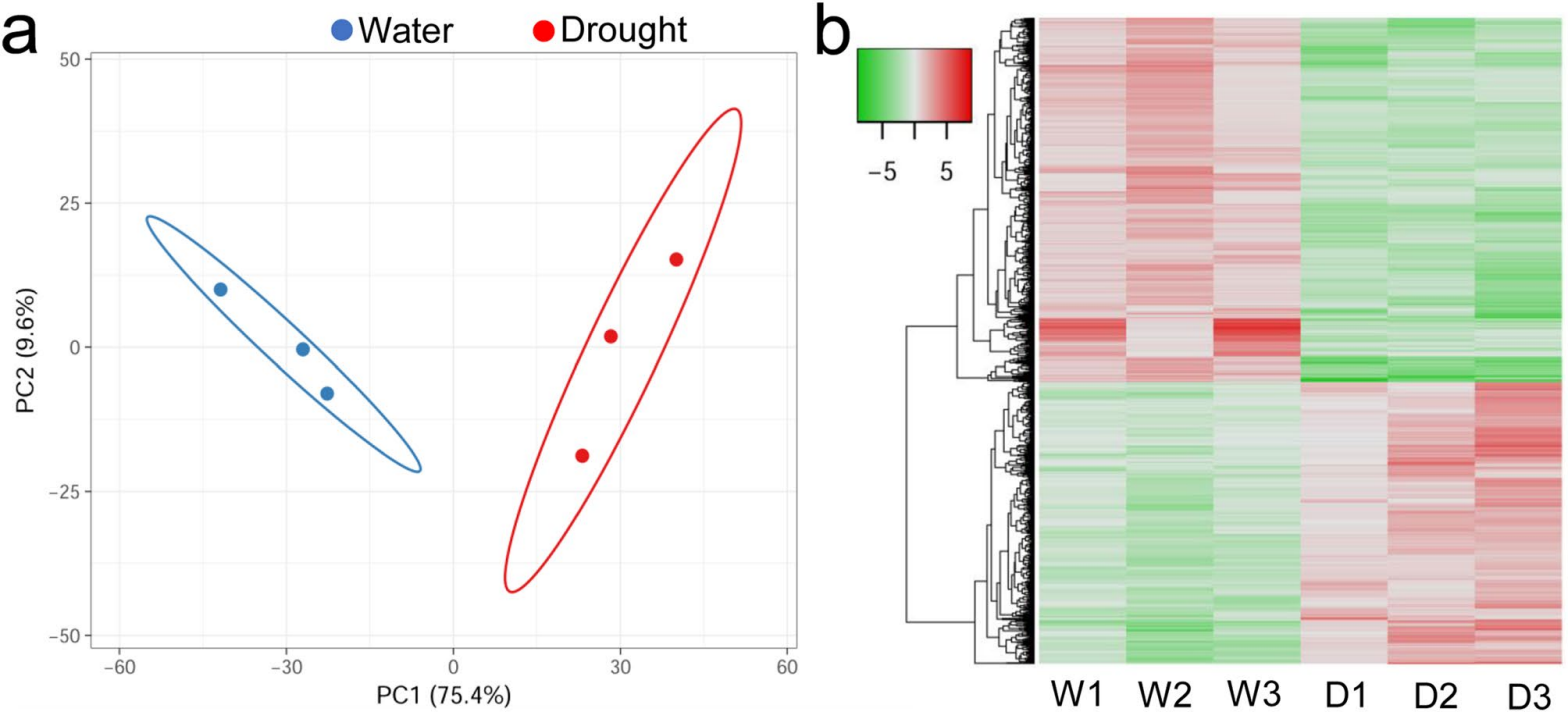
Principal component analysis (PCA) among samples of the experimental (Drought) and control (Water) groups in oil palm (**a**) and hierarchical clustering (**b**) of the relationships of samples between experimental (Drought) and control (Water) groups based on randomly selected DEGs. W1, W2 and W3 are controls, while D1, D2 and D3 are experimental samples.

A total of 2084 and 1358 DEGs were identified using two approaches: DESeq2 and EdgeR, respectively, within which 1293 were shared by the two data sets (Fig. 2). DESeq2 identified 944 down-regulated and 1140 up-regulated DEGs, while EdgeR screened 624 down-regulated and 734 up-regulated DEGs. The number of common down- and up-regulated DEGs were 614 and 679, respectively, between the two approaches (Fig. 2; Supplementary Table S2). To obtain confident results, only the common DEGS were kept for further analysis. Based on the relative expression of DEGs across samples, the drought stressed and control samples were clearly differentiated by both PCA and hierarchical clustering analyses and showed substantial differences in expression profiles (Fig. 3). We further assessed the accuracy of the RNAseq data by comparing to the results of qPCR of randomly selected nine genes (Supplementary Table S3). We observed an overall high consistency of the expression patterns of these genes between RNA-seq and qPCR (Supplementary Fig. S1a), with a correlation coefficient of 0.978 (*P* < 0.0001), as examined using Pearson’s correlation test (Supplementary Fig. S1b). Taken together, these data indicate that the RNA-seq data is reliable.

Interestingly, we observed that most of the DEGs in subcategories phenylalanine metabolism and tryptophan metabolism were down-regulated (Table 1). In plant species, phenylalanine and tryptophan metabolisms are more involved in pathogen related immune responses^40^. The down regulation of most DEGs within these categories implies the effects of metabolic compensation to drought stress responses by sacrificing the less important biological functions. In addition, we found two genes: two-component response regulator ORR9 and two-component response regulator ORR24 that were down- and up-regulated, respectively, were enriched into the subcategory: zeatin biosynthesis (Table 1), which plays important roles in drought stress response in *Populus simonii*^33^. Further studies on how the two genes are involved in the responses to drought stress in oil palm, are required.

**Table 1:** Selected enriched KEGG pathways and DEGs in response to drought challenge in the roots of oil palm seedlings.

Gene	Annotation	Subsignaling pathways	Expression
**Plant hormone signal transduction**			
LOC105046997	Jasmonic acid-amido synthetase JAR1	a-Linolenic acid metabolism	Up
LOC105048226	Jasmonic acid-amido synthetase JAR1	a-Linolenic acid metabolism	Down
LOC105051062	Protein TIFY 9	a-Linolenic acid metabolism	Up
LOC105055031	Transcription factor MYC2	a-Linolenic acid metabolism	Up
LOC105049192	Probable xyloglucan endotransglucosylase/hydrolase protein 23	Brassinosteroid biosynthesis	Down
LOC105043050	Probable protein phosphatase 2C 75	Carotenoid biosynthesis	Up
LOC105053585	Probable protein phosphatase 2C 75	Carotenoid biosynthesis	Up
LOC105056896	Probable protein phosphatase 2C 24	Carotenoid biosynthesis	Up
LOC105059101	Ethylene-responsive transcription factor 1B	Cysteine and methionine metabolism	Up
LOC105034229	Transcription factor TGAL5	Phenylalanine metabolism	Down
LOC105061166	Pathogenesis-related protein 1B	Phenylalanine metabolism	Down
LOC105061545	Pathogenesis-related protein PRB1-2	Phenylalanine metabolism	Down
LOC105061554	Pathogenesis-related protein PRB1-2	Phenylalanine metabolism	Down
LOC105042180	Auxin-responsive protein IAA30	Tryptophan metabolism	Down
LOC105046556	Auxin-responsive protein IAA30	Tryptophan metabolism	Down
LOC105047880	Auxin-responsive protein IAA1	Tryptophan metabolism	Up
LOC105051115	Indole-3-acetic acid-amido synthetase GH3.17	Tryptophan metabolism	Down
LOC105051940	Auxin transporter-like protein 3	Tryptophan metabolism	Down
LOC105055859	Auxin-responsive protein IAA30	Tryptophan metabolism	Down
LOC105056155	Probable indole-3-acetic acid-amido synthetase GH3.8	Tryptophan metabolism	Up
LOC105049107	Two-component response regulator ORR9	Zeatin biosynthesis	Down
LOC105049543	Two-component response regulator ORR24	Zeatin biosynthesis	Up
**MAPK signaling pathway**			
LOC105043050	Probable protein phosphatase 2C 75	Abscisic acid	Up
LOC105053585	Probable protein phosphatase 2C 75	Abscisic acid	Up
LOC105056896	Probable protein phosphatase 2C 24	Abscisic acid	Up
LOC105052054	Protein MKS1-like	Pathogen infection	Up
LOC105061166	Pathogenesis-related protein 1B	Pathogen infection	Down
LOC105061545	Pathogenesis-related protein PRB1-2	Pathogen infection	Down
LOC105061554	Pathogenesis-related protein PRB1-2	Pathogen infection	Down
LOC105055031	Transcription factor MYC2	Phytohomones	Up
LOC105059101	Ethylene-responsive transcription factor 1B	Phytohomones	Up
LOC105059691	Chitinase 1	Phytohomones	Up
**ABC transporters**			
LOC105043189	ABC transporter C family member 5	ABC1 subfamily	Up
LOC105032304	ABC transporter B family member 11	ABCB subfamily	Down
LOC105038824	Putative multidrug resistance protein	ABCB subfamily	Down
LOC105041030	ABC transporter B family member 9	ABCB subfamily	Up
LOC105056548	ABC transporter B family member 19	ABCB subfamily	Down
LOC105060251	Putative multidrug resistance protein	ABCB subfamily	Up

### Ontology enrichment analysis of genes responding to drought stress

**Figure 4:**
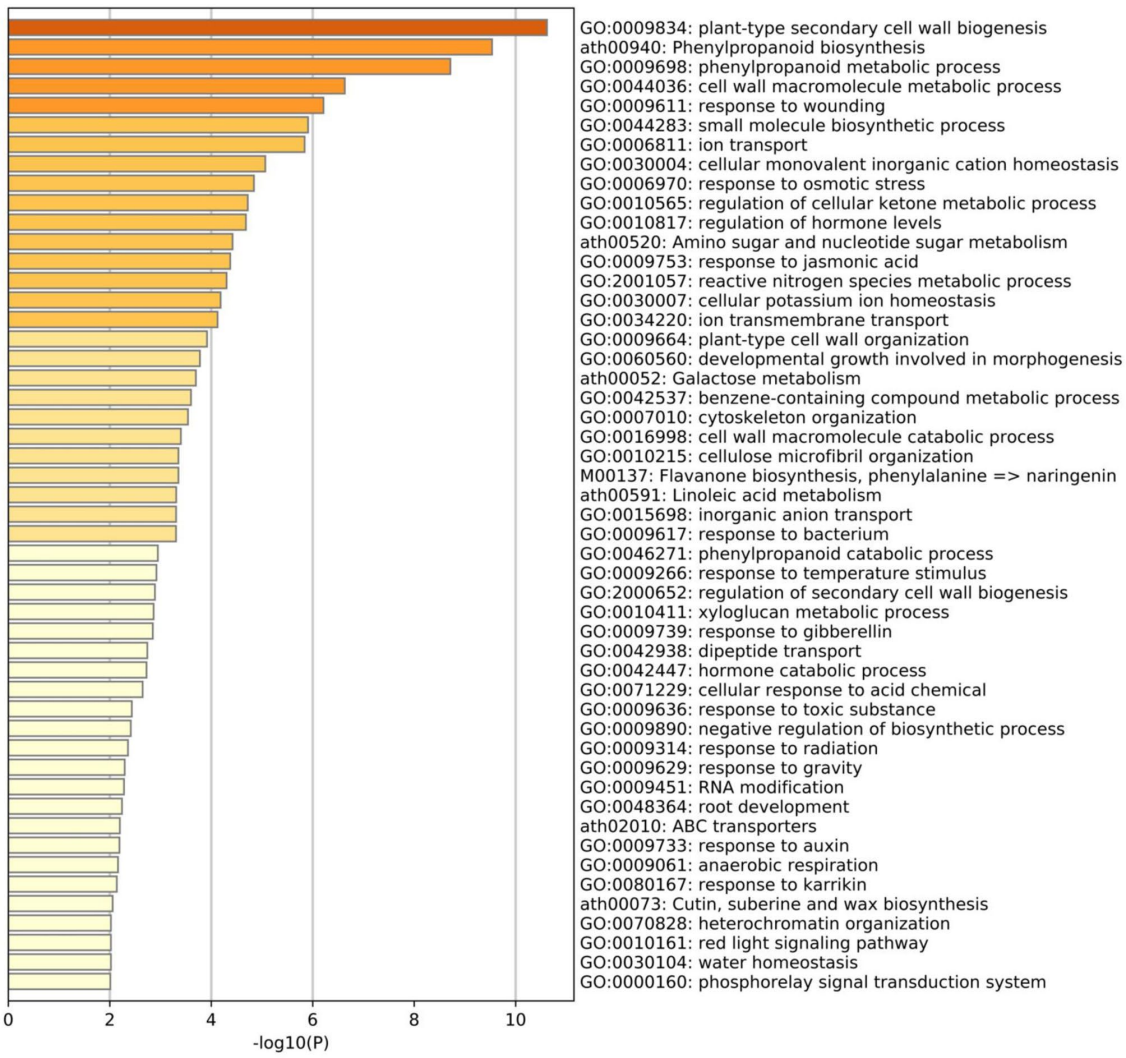
Enrichment of gene ontology (GO) of DEGs against drought challenge at the significance level of 0.01 in the roots of oil palm seedlings under drought stress.

To understand the transcriptomic responses to the drought stress, we first carried out gene ontology enrichment analysis using the 1293 DEGs identified by both EdgeR and DESeq2. A total of 89 GO terms were significantly enriched, involving many categories of diverse functions (Supplementary Table S4). The most significant enrichment entities included GO terms related to cell wall biogenesis and functions (e.g., GO:0009834, GO:0044036, GO:0009664, GO:0016998 and GO:2000652), which was consistent with the our observation that the cell wall of the drought treatments has likely been damaged by severe drought stress and thus has triggered the mechanism of damage and repair (Fig. 4). Moreover, we also observed significant enrichments related to phenylpropanoid biosynthesis and metabolisms (e.g., ath00940 and GO:0009698 and GO: 0046271). It is known that phenylpropanoid pathway is activated by stress conditions, such as drought, salinity and extreme temperature, and leads to accumulation of phenolic compounds, which play critical physiological roles in regulation under abiotic stress to cope with environmental challenges^41^. Moreover, we found some GO terms classified into the groups related to ion transport and homeostasis (e.g., GO:0006811, GO:0030004 GO:0030007, GO:0015698 and GO:0034220) and response to osmotic stress and water homeostasis (e.g., GO:0006970 and GO:0030104). Differential expressions of genes in these functions likely result from the responses of plants to water deprivation by direct regulation of osmotic pressure^42,43^. In addition, a number of genes were enriched into the biological categories related to regulation of cellular ketone metabolic process (GO:0010565), suggesting that genes involved in ketone metabolic process play important roles in drought stress in oil palm^44,45^. Hormone regulations are also indispensable to stress responses of plant species. Here, we identified two enriched GO terms related to hormone regulation and metabolism (e.g., GO:0010817 and GO:0042447). Previous studies have shown that production of numerous secondary metabolites is essential for physiological processes to respond to abiotic stress^46,47^. Consistent with these results, we found several significant enrichments related to these terms: small molecule biosynthetic process (GO:0044283), amino sugar and nucleotide sugar metabolism (ath00520), galactose metabolism (ath00052), benzene-containing compound metabolic process (GO:0042537), linoleic acid metabolism (ath00591) and xyloglucan metabolic process (GO:0010411). Interestingly, we also identified significant enriched GO terms, like response to jasmonic acid (GO:0009753) and ABC transporters (ath02010), which play crucial roles in abiotic stress responses (Fig. 4).

**Figure 5:**
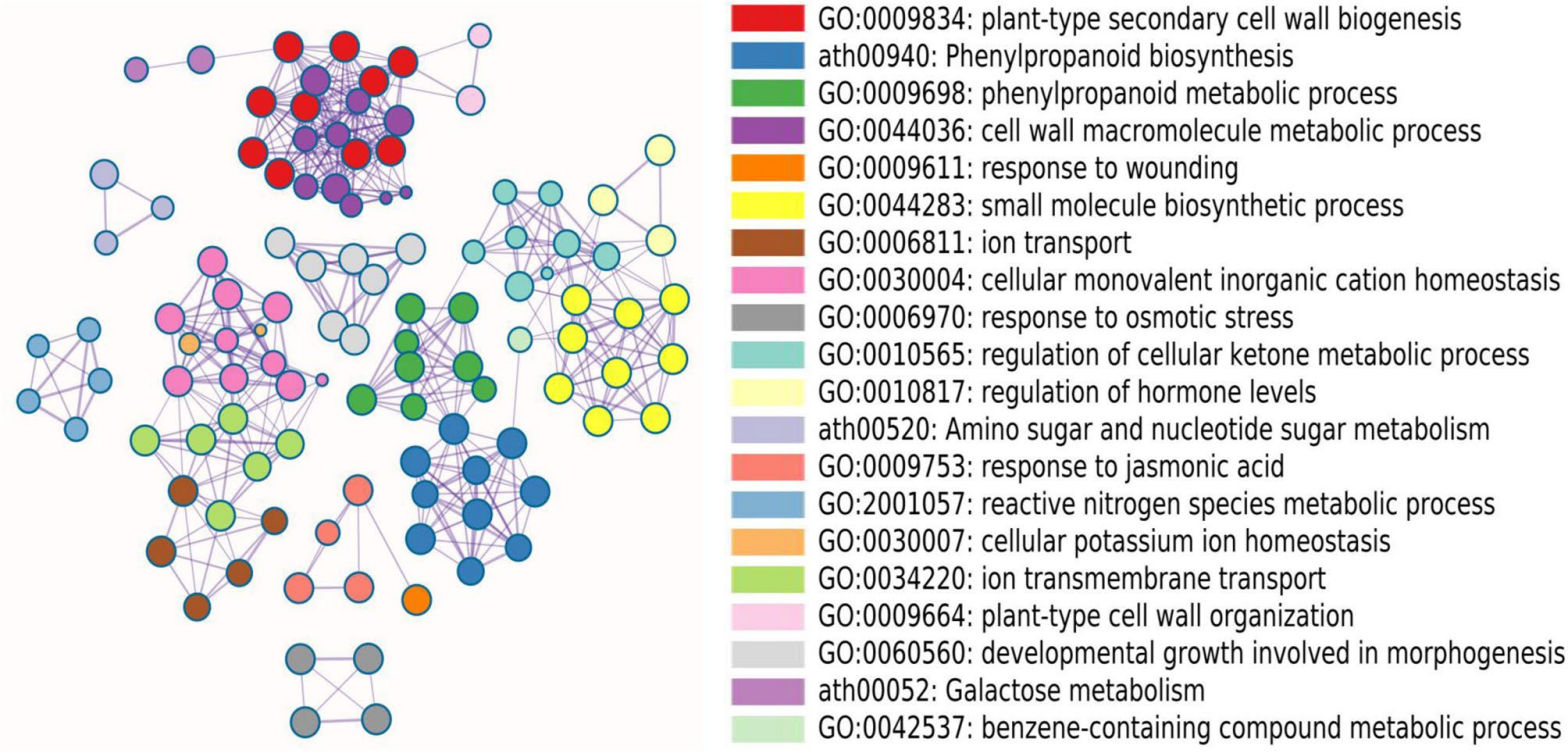
Major gene networks among the top 20 enriched GO terms as shown in this figure, based on DEGs against drought challenge in the roots of oil palm seedlings. Each network and the corresponding GO term are indicated with the same color.

The interactions of these enriched GO terms were further investigated using network analysis. Eight enriched GO networks were identified, with each consisting of no less than 3 genes (Fig. 5). The major GO networks involved those related to cell wall related biogenesis and metabolism (GO:0009834 and GO:0044036), small molecule related biosynthetic and metabolic processes (ath00940, GO:0009698, GO:0044283 and GO:0010565) and ion transport and homeostasis related processes (GO:0006811, GO:0034220 and GO:0030004). These data imply that genes in these networks are more extensively induced to differentially express to respond to drought stress^42,47,48^. We further investigated the enriched KEGG pathways and found that the functions of the enriched pathways were generally consistent with those of the enriched GO terms as shown above (Supplementary Table S5). Above all, these enrichment analyses suggest that many genes, pathways and networks respond to the drought stress in the roots of oil palm seedlings. The DEGs, pathways and networks identified in this study provide valuable resources for future studies on their functions to improve drought tolerance of oil palm.

### Plant hormone signal transduction in drought stress responses

Plant hormones not only play crucial roles in controlling growth and development, but also are indispensable in regulation of stress responses^49^. Herein, we first focused on the DEGs involved in plant hormone signal transduction pathway and found significant enrichments of DEGs within subcategories of KEGG pathways including a-Linolenic acid metabolism, carotenoid biosynthesis, phenylalanine metabolism, tryptophan metabolism and zeatin biosynthesis (Table 1). Previous studies revealed that genes related to a-Linolenic acid metabolism played important roles in drought stress responses^50,51^. We found that four DEGS were involved in a-Linolenic acid metabolism and three out of them were up-regulated. Interestingly, the down-regulated DEG, jasmonic acid-amido synthetase JAR1 (LOC105048226), was a duplicated copy of the up-regulated one, jasmonic acid-amido synthetase JAR1 (LOC105046997), suggesting functional divergence of paralogous genes since genome duplication events. Nevertheless, consistent with the expression patterns of most DEGs in this subcategory, the up-regulated jasmonic acid-amido synthetase JAR1 might be more important in regulation of drought stress response in oil palm. Moreover, we found that all of the DEGs, including probable protein phosphatase 2C 24 and two duplicated copies of probable protein phosphatase 2C 75, in the subcategory carotenoid biosynthesis, were up-regulated. These three DEGs were also enriched into the subcategory abscisic acid pathway (ABA) within MAPK signalling pathway (Table 1). Carotenoid biosynthesis signalling pathway is specifically induced by root and contributes to induce ABA production to regulate ion homeostasis, as studied in *Arabidoposis*^52^. ABA-independent signalling pathways are involved in the regulation of drought stress response in many plant species^53^. Therefore, our results suggest that ABA related genes also play important roles in drought stress responses of oil palm.

### ABC transporters in drought responses

Membrane transporters play vital roles in regulation of water and ion homeostasis of organisms, among which ATP-binding cassette (ABC) transporters constitute one of the largest protein families and act as both exporters and importers, driven by ATP hydrolysis^54^. ABC transporters play irreplaceable roles in transmembrane allocations of various molecules to adapt to rapidly changing environments, such as water scarcity, heavy metal stress and pathogen stress^55^. In order to survive in these changing abiotic conditions, it is necessary for cells to absorb nutritious chemical substances and discharge endogenous toxins, as well as exchange signalling molecules^55^. Thus, the ABC transporters occupy a diverse range of functions and hence the regulations upon stress responses are also complicated. Here, we found that six DEGs were enriched into the pathway of ABC transporters (Table 1). Five of them were ABCB subfamily members, among which three [ABC transporter B family member 11, ABC transporter B family member 19 and putative multidrug resistance protein (LOC105038824)] were down-regulated and two [ABC transporter B family member 9 and another putative multidrug resistance protein (LOC105060251)] were up-regulated. Such differential expression patterns of these ABCB subfamily transporters indicate the complicated functions in controlling of influx and efflux of chemical molecules^56,57^. In addition, we also identified an ABCC subfamily member, ABC transporter C family member 5, which was up-regulated. Interestingly, two putative multidrug resistance protein genes (LOC105038824 and LOC105060251) were differentially expressed against drought stress. As shown in previous studies, multidrug resistance‐associated proteins are widely involved in regulation of stress responses, such as salt stress, water deprivation, oxidative stress and fungal stress^58^. Taken together, these different types of ABC transporters likely play important roles in responses to drought stress in oil palm.

### Protein–protein interaction networks in response to drought responses

**Figure 6:**
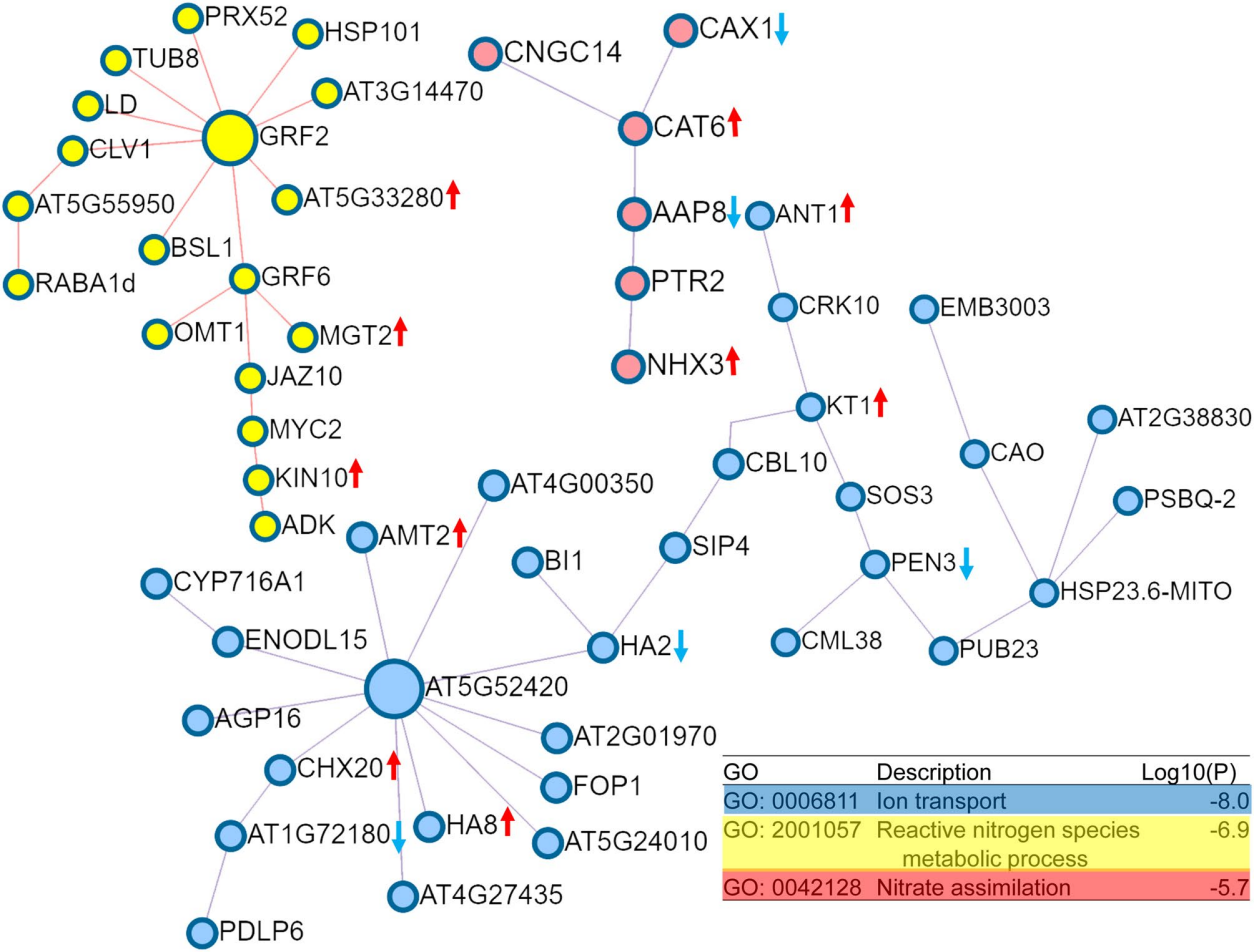
Three significant enrichments of protein–protein interaction networks identified based on DEGs against drought challenge in the roots of oil palm seedlings. DEGs in the three networks are further enriched with GO terms, with each showing corresponding significance. The networks are shown in different colors. The DEGs within each network are indicated and the expression patterns are shown with colored up- and down-arrows.

Other than significantly enriched GO terms and KEGG pathways, we also identified three protein–protein interaction networks, focused on ion transport, reactive nitrogen species metabolic process and nitrate assimilation (Fig. 6; Table 2). Eight DEGs were involved in the ion transport network, among which five [ammonium transporter 2 member 1 (AMT2-1), amino acid transporter ANT1 (ANT1), cation/H(+) antiporter 20 (CHX20), plasma membrane ATPase 4 (PMA4) and potassium channel AKT1 (AKT1)] and three [receptor-like protein kinase HSL1 (HSL1), plasma membrane ATPase (PMA) and ABC transporter G family member 42 (ABCG42)] were up- and down-regulated, respectively (Table 2). Interestingly, most of the cation channel and transporter genes were up-regulated, including ammonium transporter 2 member 1 (AMT1), amino acid transporter ANT1 (ANT1), cation/H(+) antiporter 20 (CHX20) and potassium channel AKT1 (KT1), indicating their positive effects in regulating ion homeostasis in oil palm^16^. Nevertheless, we also observed that three DEGs were down-regulated in the same network, implying both positive and negative feedback regulations are acting on this network^59^. Reactive nitrogen species metabolic process is also suggested to have critical roles in stress responses, such as drought and salinity^60^. Consistently, we identified three up-regulated genes: magnesium transporter MRS2-1 (MGT2), putative chloride channel-like protein CLC-g (AT5G33280) and serine/threonine protein kinase OSK1 (KIN10), in this protein–protein interaction network. Nitrate assimilation is another biological process affecting salt and water stress tolerance in plants^61^. Here, we found four DEGs involved in this network: two were up-regulated [cationic amino acid transporter 6, chloroplastic (CAT6) and sodium/hydrogen exchanger 4 (NHX4)], while the other two were down-regulated [amino acid permease 8 (AAP8) and vacuolar cation/proton exchanger 1a (CAX1)]. As these protein–protein interaction networks play crucial roles in drought stress response, the DEGs involved in these networks provide important candidate genes to improve drought tolerance of oil palm by genetic engineering and/or selective breeding.

**Table 2:** DEGs in three enriched protein–protein interaction networks in response to drought challenge in the roots of oil palm seedlings.

Gene	Annotation	Expression
**Ion transport**		
LOC105045862	Ammonium transporter 2 member 1	Up
LOC105049578	Amino acid transporter ANT1	Up
LOC105037902	Receptor-like protein kinase HSL1	Down
LOC105033828	Cation/H(+) antiporter 20	Up
LOC105039277	Plasma membrane ATPase	Down
LOC105052943	Plasma membrane ATPase 4	Up
LOC105047848	Potassium channel AKT1	Up
LOC105040536	ABC transporter G family member 42	Down
**Reactive nitrogen species metabolic process**		
LOC105060165	Magnesium transporter MRS2-1	Up
LOC105050121	Putative chloride channel-like protein CLC-g	Up
LOC105044259	Serine/threonine protein kinase OSK1	Up
**Nitrate assimilation**		
LOC105037451	Amino acid permease 8	Down
LOC105052094	Cationic amino acid transporter 6, chloroplastic	Up
LOC105032563	Vacuolar cation/proton exchanger 1a	Down
LOC105038946	Sodium/hydrogen exchanger 3	Up

### Transcription factors in drought responses

To date, more and more studies focused on the biological functions of transcription factors as regulatory elements binding proteins^21^. Transcription factors are vital for development, response to intercellular and environmental signals and pathogenesis^21^. The expression changes are often associated with important cellular processes^15^. In this study, we identified 96 differentially expressed transcription factors that were classified into 28 families (Supplementary Table S6, Table 3). Previous studies have shown that transcription factors are broadly involved in drought/abiotic stress responses, such as the members of family MYB, WRKY, DREB, NAC and AP2/EREBP^27,62–64^. Here we also observed that genes in these transcription factor families were differentially expressed under drought stress in oil palm, further supporting their important roles in drought tolerance in plant species. Interestingly, we found several families of transcription factors that were rarely studied and involved in abiotic stress responses, such as the C2H2, LFY and TALE transcription families. Therefore, it is important to understand the mechanisms of regulatory functions of these genes, which might be useful to help improve drought tolerance of related plant species.

**Table 3:** Categorization of differentially expressed transcription factors and the patterns of their expressions after drought stress in the roots of oil palm seedlings.

Family	Number	Down	Up
AP2	2	1	1
ARR-B	2	0	2
B3	3	0	3
bHLH	10	4	6
bZIP	5	2	3
C2H2	5	1	4
C3H	1	0	1
Dof	1	0	1
E2F/DP	1	0	1
ERF	9	1	8
FAR1	1	0	1
G2-like	2	1	1
GATA	1	0	1
GRAS	2	0	2
GRF	1	1	0
HB-other	1	0	1
HD-ZIP	6	3	3
HSF	3	0	3
LBD	1	0	1
LFY	1	0	1
MYB	15	9	6
NAC	17	9	8
RAV	1	0	1
TALE	1	0	1
TCP	1	0	1
Trihelix	1	0	1
WRKY	1	0	1
YABBY	1	0	1
Total	96	32	64

## Conclusions

We investigated transcriptomic response of root against drought stress in oil palm seedlings. We identified over 1000 DEGs responding to the drought stress, including the genes mainly involved in cell wall biogenesis and functions, phenylpropanoid biosynthesis and metabolisms and ion transport and homeostasis. We functionally enriched the genes in plant hormone signal transduction and ABC transporters pathways, which likely have played crucial roles in regulation of water deprivation. Three protein–protein interaction networks were identified that were related to ion transport, reactive nitrogen species metabolic process and nitrate assimilation. We also detected 96 transcription factors that were differentially expressed upon drought stress. The identified DEGs, pathways and protein–protein interaction networks, and transcription factors likely play important roles in drought tolerance of oil palm. This study helps understand more about the mechanism of drought stress response and provides valuable resources for future genetic improvement of drought tolerance in oil palm. Future studies should analyse the functions of DEGs identified, in combination with metabolomics and morpho-physiological approaches to obtain a comprehensive overview of drought stress impact on oil palm.

## Materials and methods

### Plant materials and drought treatment

Seeds of *Tenera* palms (*Elaeis guineensis*, Jacq.) were geminated with a standard protocol^3,53^. The seedlings were grown in a nursery for 120 days before drought stress treatment. Eight oil palm *Tenera* seedlings were planted in pots with diameter of 20 cm containing natural soil with water content of 23% (2.3 g water per 10 g soil), and placed in a greenhouse with a natural tropical temperature ranging from 28 to 34 °C, 30–50% relative humidity and natural photoperiod. Four seedlings were watered twice a week to maintain water content of > 23% while the other four seedlings were used as drought treatment without watering for two weeks. After drought stress challenge of 14 days, the mortality rate of experimental group was estimated at ~ 50%. The root tissues of both the control and experimental groups were harvested and measured, respectively. The samples were then preserved at − 80 °C for RNA isolation.

### RNA extraction and sequencing

Total RNA was isolated from roots using RNeasy Plant Mini Kit (Qiagen, Germany), according to the manufacturer’s instructions. RNA quality was assessed by agarose gels and concentration was measured by NanoDrop (Thermo Fisher Scientific, USA). One µg total RNA from each sample was firstly treated with RNase-free DNase I (Sigma-Aldrich, Singapore) and then used for mRNA library construction with Illumina TruSeq RNA Library Prep Kit v2 (Illumina, USA), according to the manufacturer’s instructions. The libraries were paired-end sequenced (2 × 75 bp) using an Illumina NextSeq500 (Illumina, USA). Three biological replicates were sequenced for both control and drought treated samples. For validation of RNA sequencing data using real-time quantitative PCR (qPCR), two µg total RNA was treated with RNase-free DNase I (Sigma-Aldrich, Singapore) and was then used for synthesizing cDNA with the MMLV reverse transcriptase (Promega, USA).

### Identification of differentially expressed genes (DEGs)

Raw sequencing reads were processed using the program *process_shortreads* in Stacks package^65^, to demultiplex samples, filter adaptors and clean up low quality reads. The program STAR^66^ was employed to align and map the cleaned reads to the reference genome of oil palm^37^, with default parameters. Only uniquely mapped reads were used to analyse the expression patterns of annotated genes. The program HTSeq-count^67^ was then used to count the expression level of each annotated gene, based on the information of gene features in the genome annotation file. We used both DESeq2^68^ and EdgeR^69^ to normalize the relative expression of transcripts across samples. Only transcripts with the number of counts per million (CPM) mapped reads of > 1 were retained for further analysis. Transcripts with a fold change (FC) value of > 2 or < − 2 and with a significance value of 0.01 after application of Benjamini–Hochberg false discovery rate (FDR)^70^ were considered as differentially expressed genes, between drought treatment and control groups. Only DEGs that were consistently identified by both DESeq2 and EdgeR, were used for further analysis.

### Functional annotation of DEGs

Gene Ontology (GO) and Kyoto Encyclopedia of Genes and Genomes (KEGG) accessions ^71^ were retrieved for each DEG, according to the PalmXplore database of oil palm^72^. We first clustered all the samples using both principal component analysis (PCA) and heatmap approaches with the program ClustVis^73^, based on the relative expression of DEGs, to investigate the overall expression patterns between drought treatment and control groups. Gene ontology enrichment analysis was carried out using the program Metascape ^74^. The Metascape program^74^ was further employed to study the protein–protein interactions using network analysis by referencing to Arabidopsis. The candidate signalling pathways associated DEGs were classified and enriched by annotating against the Kyoto Encyclopaedia of Genes and Genomes (KEGG) database^75^ of oil palm.

### Validation of RNA-seq data using qPCR

DEGs were randomly selected and the relative expression patterns revealed by RNA-seq were examined by qPCR, to assess the effectiveness and accuracy of the whole DEGs dataset. Primers of randomly selected DEGs were designed according to the coding sequences, obtained from the annotated reference genome, using the program Primer3^76^. Both β-actin gene and glyceraldehyde 3-phosphate dehydrogenase gene (GAPDH) were used as housekeeping genes to normalize the relative expression of genes, according to our previous study^77^. The 2^−ΔΔCT^ method was used to quantify the expression level according to our previous method^78^. The experiment was carried out with three biological replications, each with three technical replicates.

### Ethics declarations

All authors have reviewed the final version of the manuscript and agree to publish the data.

## Supplementary Information

Supplementary Information S1.

Supplementary Information S2.

## Data Availability

Raw sequencing reads used in this study have been deposited to the NCBI SRA database with an accession no. PRJDB9517.
