# Supplementary material for: Genes, pathways and networks responding to drought stress in oil palm roots
Source: Sci Rep. 2020 Dec 4;10:21303. doi: 10.1038/s41598-020-78297-z (PMC7719161; doi:10.1038/s41598-020-78297-z)
Supplement: Supplementary file 1 — Supplementary Information S1. [file 41598_2020_78297_MOESM1_ESM.pdf]

Supplementary Figure 1 for the paper entitled

Genes, pathways and networks responding to drought stress in oil palm roots

Le Wang[1], May Lee[1], Baoqing Ye[1] and Gen Hua Yue[1, 2#]

[1] Molecular Population Genetics and Breeding Group, Temasek Life Sciences Laboratory, 1 Research Link, National University of Singapore, Singapore 117604

[2] Department of Biological Sciences, National University of Singapore, 14 Science Drive 4, Singapore 117543

# Correspondence: Dr. G.H. Yue

Temasek Life Sciences Laboratory, 1 Research Link, National University of Singapore, 117604 Singapore; Tel: +65-68727405; Fax: +65-68727007; Email: genhua@tll.org.sg

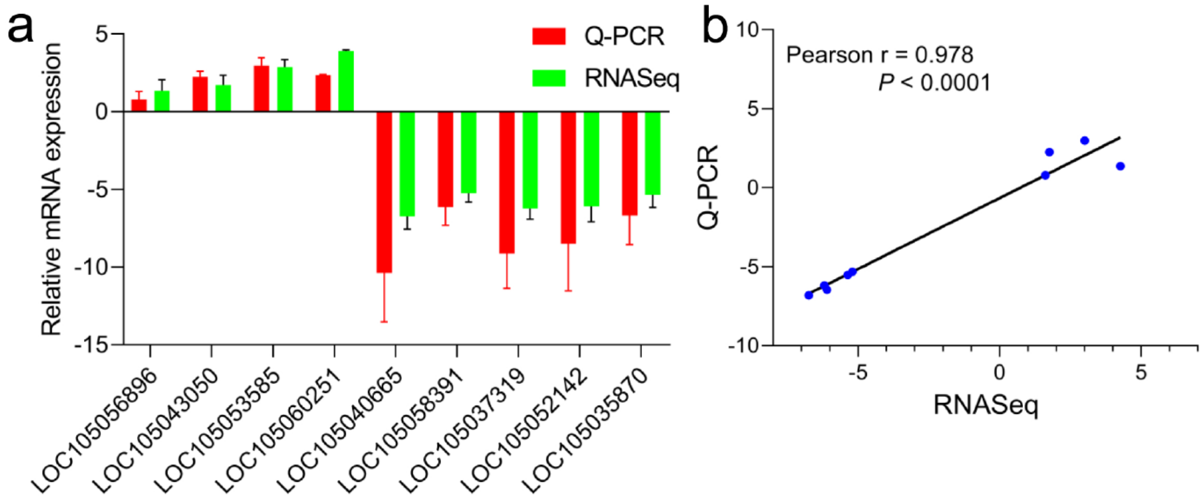

Figure S1 The relative expression of selected DEGs between real-time Q-PCR and RNA-Seq (a) and the Pearson's correlation between the two data sets (b), in the roots of oil palm seedlings.
